# A comparative study in fixation methods of medial malleolus fractures between tension bands wiring and screw fixation

**DOI:** 10.1186/s40064-016-2155-z

**Published:** 2016-04-26

**Authors:** Ayyoub A. Mohammed, Khalid Ahmed Abbas, Ammar Salah Mawlood

**Affiliations:** Department of Surgery, College of Medicine, University of Anbar, Al-Anbar, Iraq; Al-Ramadi Teaching Hospital, Al-Ramadi, Iraq; Al-Anbar Orthopedic Training Center, Al-Anbar, Iraq; Al-Yarmook Teaching Hospital, Baghdad, Iraq

**Keywords:** Ankle, Screw, Tension band, Fracture

## Abstract

**Objectives:**

The aim of this study is to compare two methods of internal fixations of fractured medial malleolus which are simple screw fixation and tension band wiring.

**Patients and methods:**

Over 5 years we grouped 20 patients with fractured medial malleolus into two groups of operative treatments, group1 treated by malleolar screw fixation and group2 by tension band wiring. The patients were with same age group, gender, fracture type, and etiology. We use statistical analysis for make a comparative study between the two ways of surgical treatment.

**Results:**

The mean time for radiologic bone union was 11.8 weeks in group1 patients and 9.4 weeks in group2 patients (P = 0.03). No patients had any sign of fixation failure or Kirschner wires migration. According to the modified ankle scoring system of Olerud and Molander, excellent and good results were achieved in 80 % in group1 patients and 90 % in group2 patients (P = 0.049).

**Conclusions:**

Tension-band wiring may be better treatment option for internal fixation of medial malleolar fractures than screw fixation.

**Recommendations:**

From these findings we recommend a further randomized clinical trial of larger number of cases and longer follow-up duration in order to regard tension-band wiring a better operative option for fixation of medial malleolar fractures.

## Background

The ankle is a close-fitting hinge of which the two parts interlock like a mortise (the box formed by the distal ends of the tibia and fibula) and tenon (the upwards projecting talus). The articulations of this joint complex are primarily between the dome of the talus and the tibial plafond, which forms highly congruent saddle-shaped weight-bearing surfaces. The talus has a medial facet, which articulates with the medial malleolus of the distal aspect of the tibia, and a lateral facet, which articulates with the lateral malleolus. Ankle fractures are among the most common musculoskeletal injuries. These injuries span a spectrum from simple closed fractures to complex open injuries. As a result, the orthopedic management is varied and can range from nonoperative casting to staged surgery with a primary focus on damage control procedures followed by definitive fixation. These fractures typically result from a low-energy indirect rotational force in which the ankle is twisted & the talus tilts &/or rotate forcefully in the mortise, causing a low-energy fracture of one or both malleoli, with or without associated ligament injuries, but can also present as a more complex, high energy injury (Solomon et al. 2010).

Nondisplaced fractures of the medial malleolus usually can be treated with cast immobilization; however, in individuals with high functional demands, internal fixation may be appropriate to hasten healing and rehabilitation. Displaced fractures of the medial malleolus should be treated surgically because persistent displacement allows the talus to tilt into varus (Michelson 2006).

## Patients and methods

From January 2010 to January 2015 at our Teaching Hospitals, we randomized 20 consecutive patients with displaced closed fractures of medial malleolus, which were isolated medial malleolus fractures or part of bi-malleolar fractures. All were treated by open reduction and internal fixation with either malleolar screw or with tension-band wiring and the choice of mode of fixation were on alternate basis. They were then allocated to one of two treatment groups:

- Group1 (10 patients) had malleolar screw fixation.
- Group2 (10 patients) had tension-band wiring.

The populations were similar in age group (median 37 years), gender, fracture type (Weber type B and C), and aetiology (twisting injury, fall, or motor vehicle accident) (Table 1).

**Table 1:** Details of 20 patients with medial malleolar fractures

	Group1 (malleolar screw)	Group2 (tension-band)
Median age in years	37 (24–50)	37 (21–53)
Male:female	4:6	4:6
Right:left	5:5	6:4
Weber B:Weber C	7:3	7:3
Causes of the fracture		
Twisting	6	6
Fall	2	3
Motor cycle accident	2	1

The fractures were classified according to the Danis–Weber classification. We exclude those with vertical fractures of medial malleolus because these fractures usually require horizontally directed screws and difficult to be fixed internally by tension-band wiring (Pankovich 2002).

Inclusion criteria:

1. Patient age 15–60 years.
2. Patient of Weber type B&C.
3. Surgical treatment.
4. Follow up for 6 months.

Exclusion criteria:

1. Age <15 years.
2. Age >60 years.
3. Patient of Weber type A.
4. Patient diagnosed and treated by Doctors other than the authors.
5. Follow up <6 months.
6. Open fractures
7. Pathological fractures
8. Fracture with incomplete treatment by the department.
9. Incomplete follow up.
10. Insufficient clinical data of the case.

### Preoperative planning

Preoperative evaluation includes assessment of general health and a thorough assessment of neurovascular status of the lower extremity. Radiographic evaluation includes anteroposterior, mortise, and lateral views of the ankle. The surgery was performed before the ankle swells up or when the swelling subsided, which was usually after 5–10 days of elevation.

### Surgical technique

All the patients in this study were operated upon under general anesthesia. The patient was positioned supine and an Esmarch or a pneumatic tourniquet was applied to the mid thigh. After routine skin preparation and draping, we made an anteromedial incision that began approximately 2 cm proximal to the fracture line, extended distally and slightly posteriorly, and ended approximately 2 cm distal to the tip of the medial malleolus. We prefer this incision for two reasons: first, the tibialis posterior tendon and its sheath are less likely to be damaged, and second, the surgeon able to see the articular surfaces, especially the anteromedial aspect of the joint, which permits accurate alignment of the fracture. Handling the skin with care and reflecting the flap intact with its underlying subcutaneous tissue. The blood supply to the skin of this area is poor, and careful handling is necessary to prevent skin sloughing. We protect the great saphenous vein and its accompanying nerve. A small fold of periosteum commonly is interposed between the fracture surfaces. We removed this fold from the fracture site with a curet or periosteal elevator, exposing the small serrations of the fracture. We debrided small, loose osseous or chondral fragments; large osteochondral fragments were preserved. With a small bone-holding clamp, the displaced malleolus was brought into normal position and, while holding it there, internally fixed with either malleolar screw or tension-band wiring.

In *group1* patients a 3.2-mm hole was drilled in a superior posterior direction while distal fragment was held reduced with a pointed clamp or with two Kirschner wires bent to stay out of way as temporary fixation devices. Length of hole was measured, and a malleolar screw was inserted without tapping till it reached the other cortex. Kirschner wires were removed after screw was tightened. In two cases the fragments were large and tend to rotate, so we used additional point of fixation (a second screw or Kirschner wire).

In *group2* patients the fracture was internally fixed with two 2-mm smooth Kirschner wires drilled perpendicular to the fracture line. The Kirschner wires should be parallel, and their ends were bent at 90° angles. This will eventually prevent the figure-of-eight wire from slipping over the exposed ends of the Kirschner wires. A stainless steel 1.2-mm AO wire was passed through the previously drilled hole and around the bent ends of the Kirschner wires in a figure-of-eight configuration. The wire was then tightened.

We were carefully inspected the interior of the joint, particularly at the superomedial corner, to make sure that Kirschner wires or the screw had not crossed the articular surfaces. In conditions were image intensifier was available, we made roentgenograms to verify the position of the screw or the Kirschner wires and any faulty insertion could be avoided. Screen control was used in 3 cases only in our study. At the end of operation we deflated the tourniquet, obtained haemostasis, and closed the wound with interrupted suture. We avoided tight stitches to prevent necrosis of the skin edges. We applied thick padding and a posterior plaster splint with the ankle in neutral position.

#### After treatment

- The ankle is immobilized in a posterior plaster splint with the ankle in neutral position and elevated.
- Postoperative X-ray was taken with anterior, lateral, and mortise views.

### Follow up

All the patients were reviewed at 10–14 days, 6 weeks, 3, and 6 months after operation. At each assessment we perform a physical examination and X-ray was taken soon after the operation, at 6 weeks, and during subsequent visits to assess radiological healing.

After 10–14 days the stitches were removed and the wound examined and any complication was reported and treated accordingly. The posterior plaster splint was changed and the patient was instructed to remove it every day and to start range-of-motion exercises.

Weight bearing was restricted for 6 weeks, after which the splint discarded and partial weight bearing started. Full weight bearing is allowed after 12 weeks.

### Evaluation

We evaluate all the patients clinically, radiologically, and functionally using a modification of the scoring system proposed by Olerud and Molander (1984) (Table 2). The scores for each component of this scale were assessed by use of a questionnaire, in combination with clinical objective criteria. The scoring scale has a maximum of 100 points (>91 excellent results, 81–90 good results, 71–80 fair results, <70 poor results) (Laarhoven et al. 1996).

**Table 2:** The modified ankle score of Olerud and Molander

Parameter	Score
Pain	
None	25
Minor (weather-dependent)	20
During sports	15
During walking on smooth surfaces	5
Constant and severe	0
Stiffness	
None	10
In the morning	5
Constant	0
Swelling	
None	10
Only in the evening	5
Constant	0
Stair-climbing	
No problem	10
Impaired	5
Impossible	0
Sports	
Normal	10
Impaired	5
Impossible	0
Supports	
None	10
Tape or wrap	5
Stick or crutch	0
Daily activity & work	
Unchanged level	25
Reduced	20
Change of job	10
Severely impaired	0
Total	100

The continuous variables were analysed between groups using Independent sample t test. P value of <0.05 was considered statistically significant.

## Results

There were no significant differences between the two groups in age (median 37 years), gender, fracture type (Weber type B and C), and aetiology (twisting, fall, or motor vehicle accident).

Review of postoperative radiographs confirmed anatomic reduction with stable fixation in all twenty patients. All the series of radiographs showed normal fracture healing and no patient had malunion, nonunion, or loss of reduction.

**Fig. 1:**
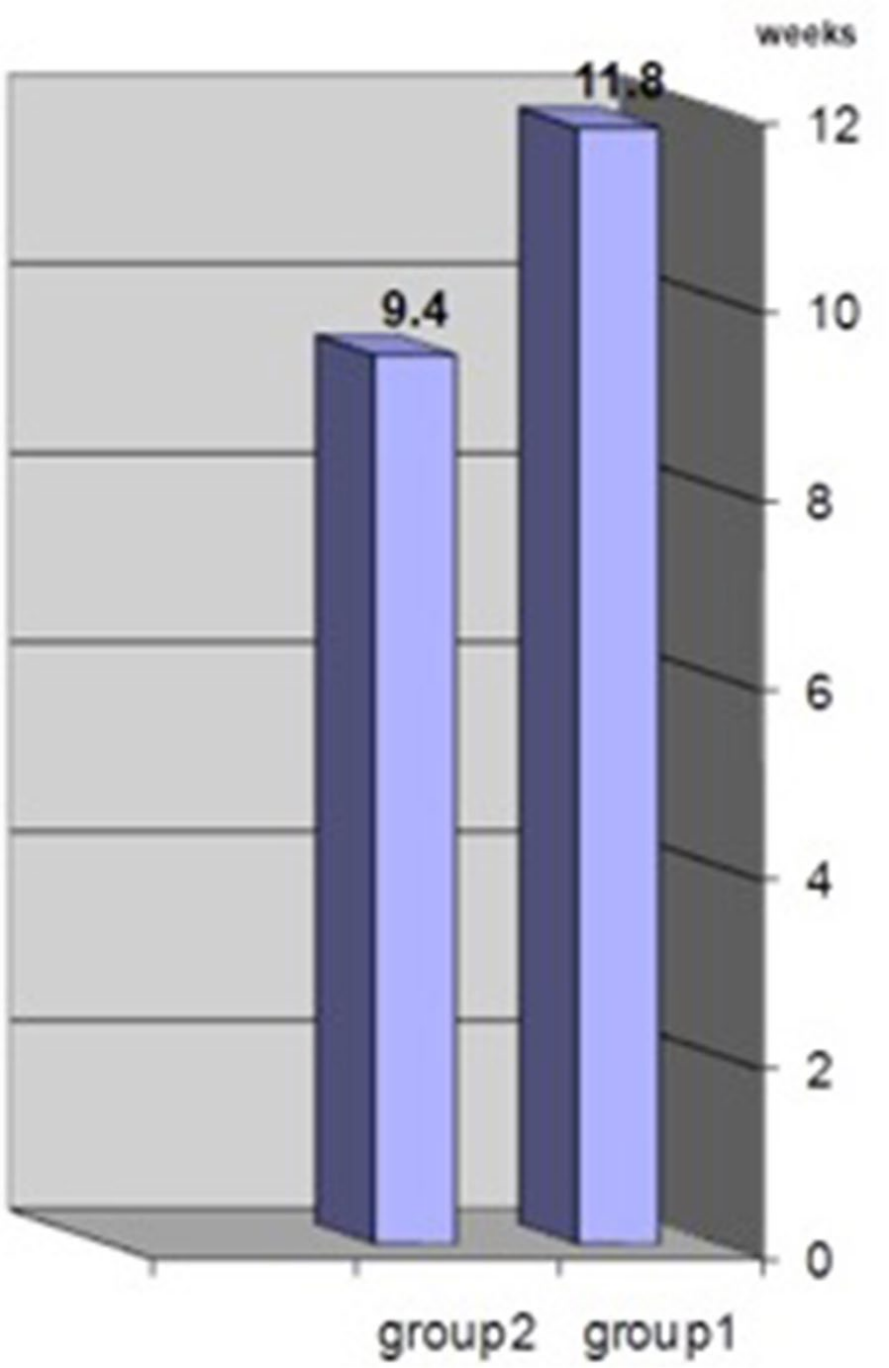
Mean time of radiologic union

The mean time for radiologic bone union was 11.8 weeks (ranging from 8 to 18 weeks) in group1 patients and 9.4 weeks (ranging from 6 to 12 weeks) in group2 patients (P = 0.03) (Fig. 1).

**Fig. 2:**
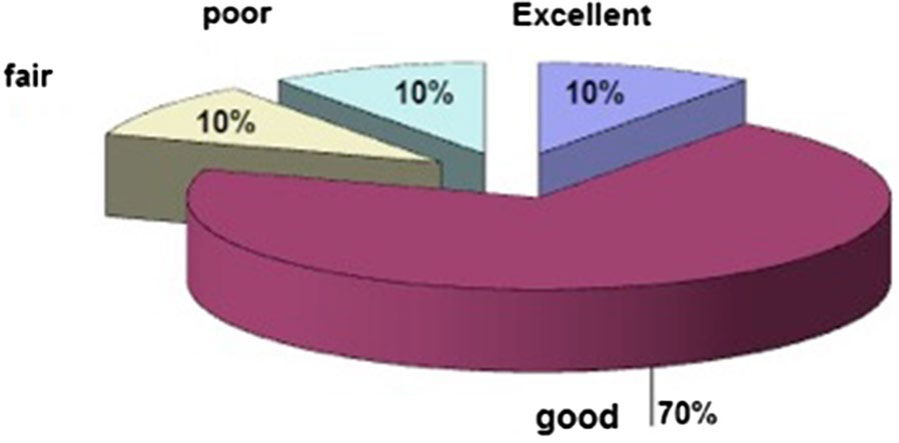
The scoring results for group1 patients

**Fig. 3:**
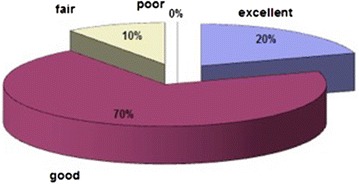
The scoring results for group2 patients

No patients had any sign of fixation failure or Kirschner wires migration. According to the modified ankle scoring system of Olerud and Molander (1984), 1 (10 %) patient in group1 and 2 (20 %) patients in group2 were excellent: good in 7 (70 %) patients in group1 and 7 (70 %) in group2: fair in 1 (10 %) patients in group1 and 1 (10 %) in group2: poor in 1 (10 %) patients in group1 and non in group2 patients (Figs. 2, 3).

**Fig. 4:**
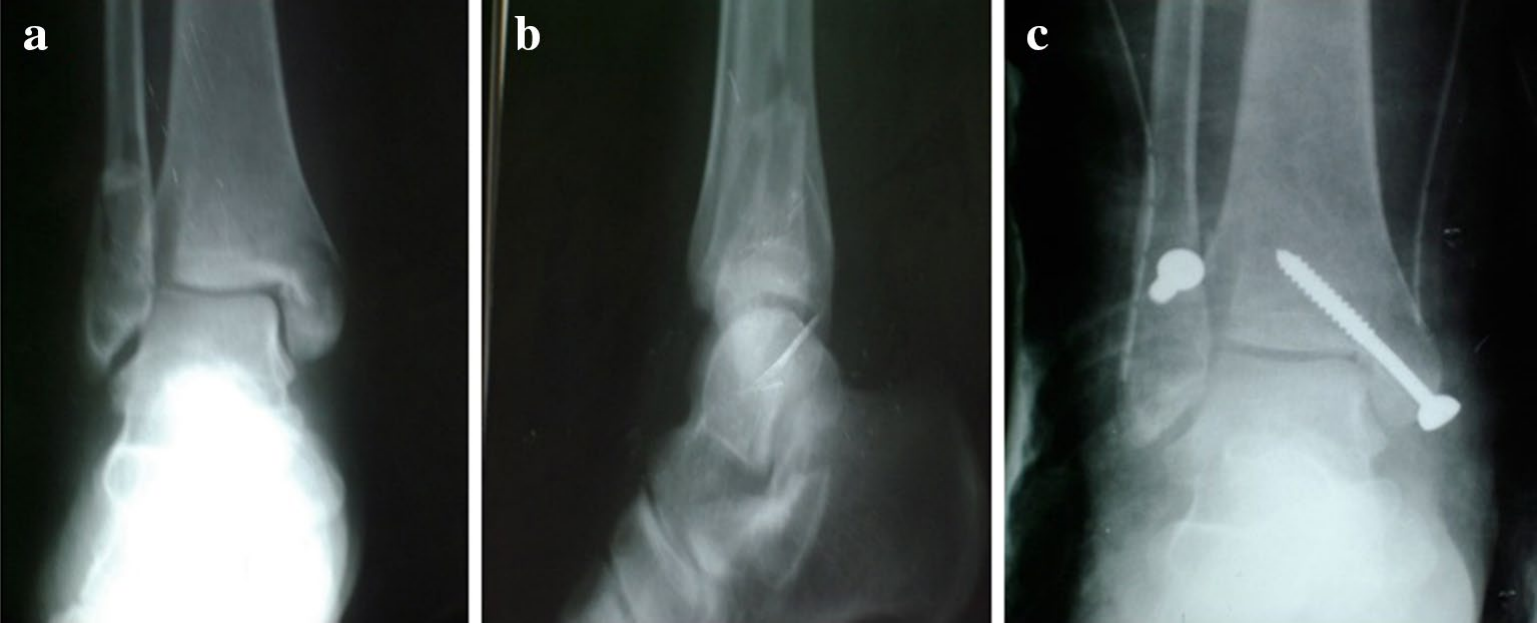
X-ray of patient in group1 treated with malleolar screw (**a** preoperative AP view, **b** preoperative lateral view, **c** first postoperative day)

**Fig. 5:**
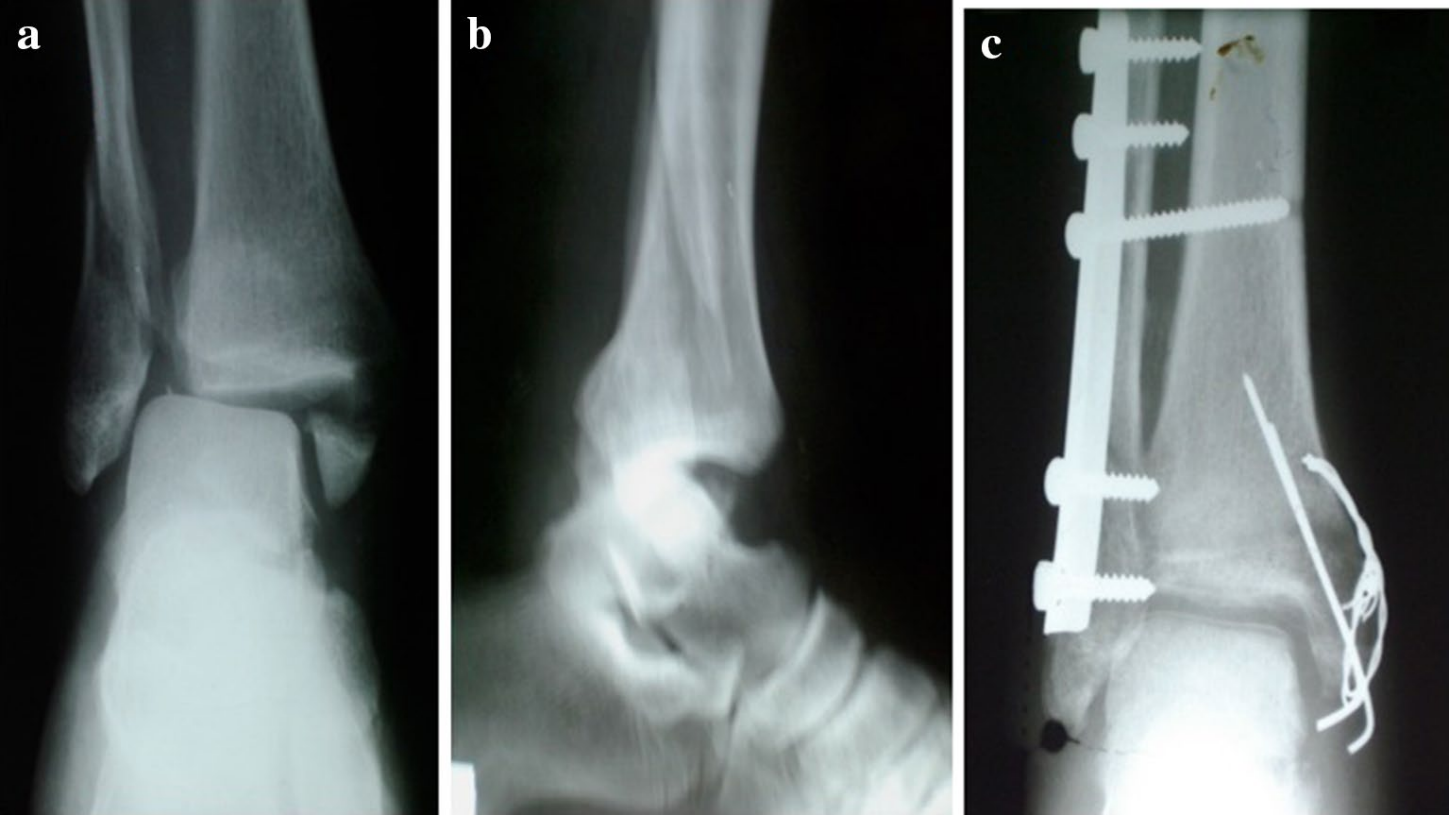
Pre- and post-operative X-rays of patient in group2 treated with tension-band wiring (**a** preoperative AP view, **b** preoperative lateral view, **c** 12 weeks postoperative)

Excellent and good results were achieved in 80 % in group1 patients (Fig. 4) and 90 % in group2 patients (Fig. 5) (P = 0.049).

## Discussion

Even though many reports of operative treatment of medial malleolar fractures have been published, comparison of the reports is difficult largely because of lack of uniformity in the subject material and in the criteria to assess the results.

According to the modified ankle scoring system of Olerud and Molander (1984), the current study showed that excellent and good results were achieved in 80 % in group1 patients (treated with malleolar screws) and 90 % in group2 patients (treated with tension-band wiring) (the difference was significant P = 0.049). This agrees with the results of Sang-Hanko and Young-Junpark who was achieved excellent and good results in about 78 % of cases treated with malleolar screws and 89 % of cases treated with tension-band wiring (Sang-Hanko and Young-JunPark 2002).

In our study the mean time for radiologic bone union was 11.8 weeks (ranging from 8 to 18 weeks) for group1 patients and 9.4 weeks (ranging from 6 to 12 weeks) for group2 patients (significant difference P = 0.03). This is similar to SK. Nurul Alam study that was reported a meantime of 12 weeks for malleolar screws and 9 weeks for tension-band wiring (Nurul Alam et al. 2007).

We have experienced only one case of delayed union (5 %) out of 20 cases of the study and no non-union developed. This case was a 50 years old female treated with malleolar screw fixation and the fracture took around 18 weeks to unite. This result slightly differs from the results of SK. Nuru Alam who was achieved 100 % union rate in both groups without any case of delayed union (Nurul Alam et al. 2007).

The low incidence of delayed union and non-union in our study might be attributed to stable anatomic reduction and limited soft tissue stripping, or due to small number of cases. Some authors reported loss of reduction with the use of tension-band technique as a result of K. wires become loose end and migrate proximally (Mack and Szabo 2005). On the other hand many authors did not agree with the frequency of this complication and reported that with the proper surgical techniques, wire migration was not a problem (Kinik and Mergen 1999). In our study we did not see any wire migration or loss of reduction. Tension-band fixation of the medial malleolar fractures has been described or referred to previously by many authors (Muller et al. 2000; Finsen et al. 1989). Ostrum and Litski recently demonstrated the biomechanics advantages of the tension-band over other fixation techniques for medial malleolus. When resisting pronation forces and applying compression force tension-band were four times stronger than malleolar screw (Ostrum and Listsky 2008). This might explain the faster union rate we were achieved in group2 patients (mean of 9.4 weeks) as compared with group1 patients (mean of 11.8 weeks). Rovinsky in his study showed that the tension-band is more technically advantageous over other types of fixation for fixation of small fragment fracture of medial malleolus and is not recommended for the fixation of vertical fracture (Rovinsky et al. 2000). We agree with these results as in our study we fixed few vertical fractures with horizontally directed malleolar screws but we excluded them from the comparison groups. Screw fixation alone may provide poor stability against torsion forces (Savage et al. 2009; Kim et al. 2005). This may requires an additional point of fixation, which may be a second screw or a Kirschner wire. Dr. Jones in his study disagrees with these results and showed that single screw fixation had similar results to double screw fixation (Jones 1997). In the current study we use additional point of fixation (second screw and K wire) in two cases in which the fragment was large and tend to rotate as screw fixation alone may provide poor stability against torsion forces.

Limitation of movements and swelling of the ankle are usually the result of neglect in treatment of soft tissue. Although better range of motion was noticed in group1 patients (80 %) as compared with group2 (70 %), it did not reach significance (P = 0.628). This could be attributed to wide soft tissue dissection that was needed with the use of tension-band. These results may show similarity with the results of SK. Nurul Alam who reported in his study that the group treated with malleolar screw showed better range of motion (Nurul Alam et al. 2007).

During surgical fixation of medial malleolar fractures excessive pressure with bone clamps to hold the fracture reduction must be avoided to prevent crushing of the fragment particularly if the bone is osteoporotic. The fracture reduction instead can hold temporarily with K wire. Careful and meticulous soft tissue handling is important for prevention of postoperative wound complications, delayed union, and, joint stiffness. We did not face any case of osteoarthritis during the follow-up period. The reason could be explained by the fact that the anatomic reduction was stable and no non-union or it may be due to short follow-up period.

## Conclusions

1. The tension-band wiring is more technically advantageous for small fragment fixation of medial malleolar fractures.
2. The tension-band wiring may be more available and its usage could translate into over all cost saving when applied to the large number of ankle fractures treated surgically in our country.
3. The faster radiological union which was achieved with the use of tension-band wiring as compared with malleolar screw could be added to the over all advantages of using this technique.

## Recommendations

These findings will have to be supported by a randomized clinical trial of larger number of cases and longer follow-up duration before recommending the tension-band wiring over malleolar screw for fixation of medial malleolar fractures.
